# Primary Osteosarcoma of the Breast: A Case Report

**DOI:** 10.1155/2013/858705

**Published:** 2013-04-07

**Authors:** Anna Rizzi, Alberto Soregaroli, Claudia Zambelli, Fausto Zorzi, Stefano Mutti, Claudio Codignola, Paola Bertocchi, Alberto Zaniboni

**Affiliations:** ^1^Medical Oncology, Fondazione Poliambulanza, Via Bissolati 57, 25124 Brescia, Italy; ^2^Radiology Department, Fondazione Poliambulanza, Via Bissolati 57, 25124 Brescia, Italy; ^3^Pathology Department, Fondazione Poliambulanza, Via Bissolati 57, 25124 Brescia, Italy; ^4^Surgical Department, Fondazione Poliambulanza, Via Bissolati 57, 25124 Brescia, Italy

## Abstract

*Introduction*. Primary osteosarcoma of the breast is a rare soft-tissue form of osteosarcoma without involvement of the skeletal system. Due to the rarity of the disease, its clinical features and optimal treatment remain unclear. *Case Presentation*. This case report deals with a 62-year-old woman with pure osteosarcoma of the breast. *Conclusions*. The prognosis of primary osteosarcoma of the breast is poor. Recurrence is frequent, and it is often associated with haematogenous spread of the disease to the lung. Treatment follows the model of sarcomas affecting other locations and must be planned in a multidisciplinary fashion. Adjuvant chemotherapy should be considered for patients with tumors showing aggressive features.

## 1. Introduction

Sarcomas of the breast are heterogeneous neoplasms derived from nonepithelial elements of the gland, and they represent less than 1% of breast cancers and less than 5% of all sarcomas [1]. 

Primary osteosarcoma of the breast is infrequently reported. In fact, although osteosarcomas constitute a common histology after breast radiation therapy, they arise mostly from adjacent bone structures (sternum, ribs) and therefore do not represent primary breast sarcomas [2].

Because of the rarity of the disease, both clinical features and optimal treatment are still to be defined.

In contrast to skeletal osteosarcoma affecting mainly young patients, primary osteosarcoma of the breast occurs in older patients, with a mean age at presentation around 65 [3].

Although there are other case reports concerning primary osteosarcoma of the breast, we believe that each case can contribute to improve the management of this rare disease.

## 2. Case Presentation

A 62-year-old Caucasian woman with a medical history significant only for hypertension presented with a self-palpated mass in her left breast.

Clinical examination showed an enlarged mass localized at the 1 o'clock position in the superior-outer quadrant of the left breast.

**Figure 1:**
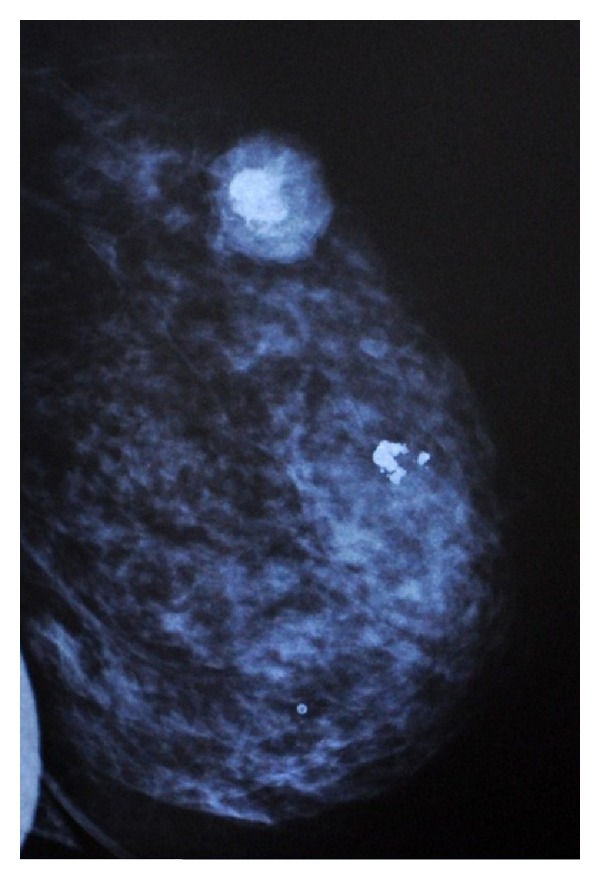
Mammographic features of the lesion.

Diagnostic mammography described a 3.7 cm × 3.6 cm mass with gross calcification in the centre and a cluster of spotty calcification around (Figure 1). No clinically enlarged nodes were detected in axilla.

High resolution ultrasonography demonstrated a densely shadowing mass, compatible with the extensive calcification identified on mammography.

Doppler evaluation showed marked vascularity in the surrounding tissue.

Cytological (FNA) examination shows neoplastic cells (C5 according to the European classification scheme); the cells were negative for epithelial markers (CAM5.2, CK AE1/AE3, CK 34*β*E12) on immunocytochemistry. Staging CT scan and skeletal scintigraphy showed no evidence of metastatic disease.

Laboratory findings were normal, including the preoperative alkaline phosphatase.

The patient underwent a left superior-outer quadrantectomy with a complete axillary dissection, as for patient's preference. Macroscopic examination of the surgical specimen revealed a well-circumscribed tumour (4,2 × 3 cm) brown in color and hard in consistency, with areas of calcification. 

**Figure 2:**
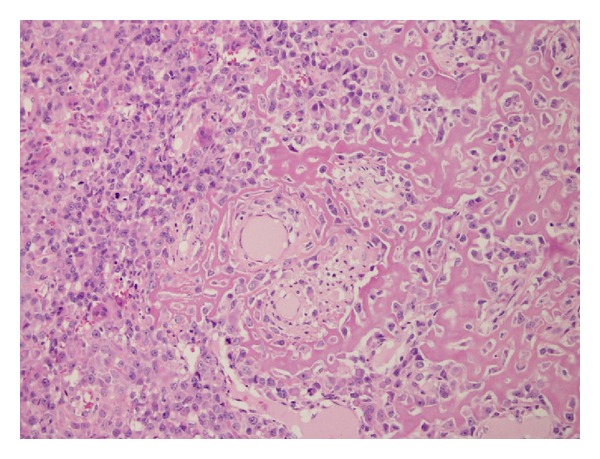
Tumor cells associated with band of osteoid with osteoblastic cells (hematoxylin-eosin 20x).

**Figure 3:**
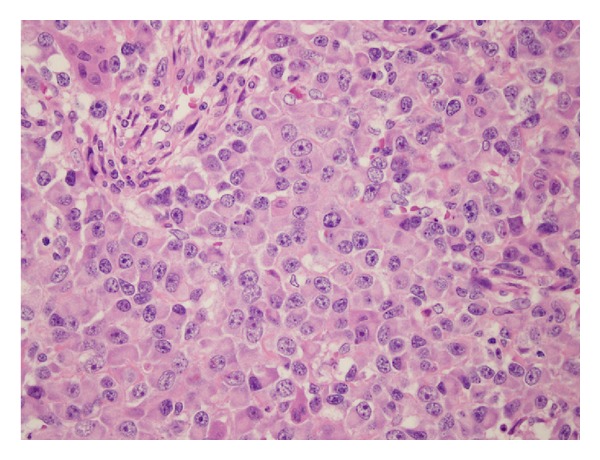
Malignant epithelioid cells and rare osteoblastic cells (hematoxylin-eosin 20x).

Microscopically, the tumour was composed of a diffuse proliferation of polygonal cells with round nuclei, evident nucleoli, and an elevated mitotic index (50 mitoses/10 HPF). Multinucleated giant cells resembling osteoclasts were interdispersed. Tumour cells produced osteoid and prominent woven bone and were supported by a diffuse network of reticulin fibres (Figures 2 and 3).

Neither lymphatic nor venous invasion was observed.

Eleven axillary lymph nodes were negative. On immunohistochemistry neoplastic cells were negative for epithelial markers (CAM 5.2, CK AE1/AE3, CK 34*β*E12, EMA) as well as for desmin, *α*-smooth-muscle actin, myogenin, and S100.

The tumour was negative for both estrogen and progesteron receptors.

A diagnosis of primitive osteoblastic osteosarcoma of the breast was formulated. The patient then received four cycles of adjuvant chemotherapy with Ifosfamide and Adriamycin and currently shows no evidence of disease (NED) after thirteen months.

## 3. Discussion

Osteosarcoma of the breast is an extremely rare tumour which should be differentiated from other two similar entities, cystosarcoma phyllodes and metaplastic carcinoma. It is possible to recognize the former by specific morphological features and the latter by the presence of carcinomatous component or cytokeratin immunopositivity on hematoxylin and eosin sections [7]. 

Osteosarcomas of the breast may arise either from pre-existing benign or malignant breast neoplasms or from previously normal breast tissue, and the histogenesis of primary osteosarcomas of the breast is unknown. Risk factors have been identified for some extraskeletal osteosarcomas and include prior local irradiation, trauma, and a foreign body. The present patient had no history of trauma or irradiation and did not have a biphasic tumor, in which case it has been suggested that carcinogenesis could be due to transformation of totipotent mesenchymal cells of the breast stroma.

A preoperative diagnosis is unusual and most patients have a correct diagnosis only after histological examination of the surgical specimen. Mammographic findings generally consist of large masses with well-defined margins and lobulated borders, which often contain coarse or dense calcifications as in fibroadenomas. A definitive diagnosis can only be made when an osteogenic sarcomas arising from the underlying bones is excluded, and immunohistochemical tests show positivity for vimentin with absence of epithelial, neural, muscular, and other markers [4].

Due to the rarity of primary osteosarcoma of the breast, there are no validated guidelines for treatment, and the best therapeutic approach remains wide local excision or mastectomy, depending on the size of the tumour and the remaining breast tissue. A complete resection with negative resection margins is needed, as margin involvement is a major predictor for local disease recurrence.

Prognostic factors for primary osteosarcomas of the breast include tumor size, number of mitoses, and presence of stromal atypia [7]. In general, osteosarcomas are aggressive tumours with blood-borne spread more common than lymphatic spread. For this reason lymphatic axillary dissection is not considered as a mainstay of surgical treatment, and a diagnosis of metaplastic carcinoma should be considered in the presence of lymph nodes metastases [5].

There is little evidence on the long-term prognosis of the disease due to the small number of cases reported in the literature. In a study of 50 patients with primary breast osteosarcoma, Silver and Tavassoli reported a 5-year survival of 38%, with 28% percent of patients developing local recurrence and 41% distant metastases [8]. Hematogenous metastases most commonly occurred in the lungs (80%), bone (20%), and liver (17%).

Indications for adjuvant chemotherapy and radiation therapy, in the absence of specific data on breast sarcoma, should follow those for soft-tissue sarcomas in general [9]. The role of postoperative radiotherapy and chemotherapy in curatively resected soft-tissue sarcomas is still controversial. Whether adjuvant radiotherapy should be used remains unclear, although several studies reporting on a small number of patients suggest that adjuvant chemotherapy may be of value in patient management [6] (Table 1).

## Figures and Tables

**Table 1:** Some case reports reported in our paper.

Author	Age	Size	Surgery	Radio Y/N	Chemo Y/N	Histology	LR*	DR^†^	Outcome
Vorobiof et al. [3]	69	3 cm	Mastectomy	N	N	Osteosarcoma	Uk	Uk	Lost
Ogundiran et al. [4]	40	18 × 20 cm	Mastectomy	Refused	N	Osteosarcoma	Uk	Nodes	6 mo
Khan et al. [5]	66	2 cm	Mastectomy	N	N	Osteosarcoma	No	No	8 y
Singhal et al. [6]	40	5 cm	Mastectomy	N	N	Osteosarcoma	No	No	5 y

*Local recurrence.

^†^Distant recurrence.

## References

[1] Voutsadakis IA, Zaman K, Leyvraz S (2011). Breast sarcomas: current and future perspectives. *Breast*.

[2] Kirova YM, Vilcoq JR, Asselain B, Sastre-Garau X, Fourquet A (2005). Radiation-induced sarcomas after radiotherapy for breast carcinoma: a large-scale single-institution review. *Cancer*.

[3] Vorobiof G, Hariparsad G, Freinkel W, Said H, Vorobiof DA (2003). Primary osteosarcoma of the breast: a case report. *Breast Journal*.

[4] Ogundiran TO, Ademola SA, Oluwatosin OM, Akang EE, Adebamowo CA (2006). Primary osteogenic sarcoma of the breast. *World Journal of Surgical Oncology*.

[5] Khan S, Griffiths EA, Shah N, Ravi S (2008). Primary osteogenic sarcoma of the breast: a case report. *Cases Journal*.

[6] Singhal V, Chintamani, Cosgrove JM (2011). Osteogenic sarcoma of the breast arising in a cystosarcoma phyllodes: a case report and review of the literature. *Journal of Medical Case Reports*.

[7] Adem C, Reynolds C, Ingle JN, Nascimento AG (2004). Primary breast sarcoma: clinicopathologic series from the Mayo Clinic and review of the literature. *British Journal of Cancer*.

[8] Silver SA, Tavassoli FA (1999). Osteosarcomatous differentiation in phyllodes tumors. *American Journal of Surgical Pathology*.

[9] Barrow BJ, Janjan NA, Gutman H (1999). Role of radiotherapy in sarcoma of the breast—a retrospective review of the M.D. Anderson experience. *Radiotherapy and Oncology*.

